# Targeting tumor-associated macrophages for cancer treatment

**DOI:** 10.1186/s13578-022-00823-5

**Published:** 2022-06-07

**Authors:** Mengjun Li, Linye He, Jing Zhu, Peng Zhang, Shufang Liang

**Affiliations:** 1 State Key Laboratory of Biotherapy and Cancer Center, West China Hospital, Sichuan University, No.17, 3rd Section of People’s South Road, 610041 Chengdu, China; 2 Department of Thyroid and Parathyroid Surgery, West China Hospital, Sichuan University, Chengdu, 610041 China; 3 Department of Urinary Surgery, West China Hospital, Sichuan University, Chengdu, 610041 China

**Keywords:** Tumor-associated macrophages, Tumor microenvironment, TAM-targeting therapy, Anticancer therapies, Immunotherapy

## Abstract

Tumor-associated macrophages (TAMs) are abundant, nearly accounting for 30–50% of stromal cells in the tumor microenvironment. TAMs exhibit an immunosuppressive M2-like phenotype in advanced cancer, which plays a crucial role in tumor growth, invasion and migration, angiogenesis and immunosuppression. Consequently, the TAM-targeting therapies are particularly of significance in anti-cancer strategies. The application of TAMs as anti-cancer targets is expected to break through traditional tumor-associated therapies and achieves favorable clinical effect. However, the heterogeneity of TAMs makes the strategy of targeting TAMs variable and uncertain. Discovering the subset specificity of TAMs might be a future option for targeting TAMs therapy. Herein, the review focuses on highlighting the different modalities to modulate TAM’s functions, including promoting the phagocytosis of TAMs, TAMs depletion, blocking TAMs recruitment, TAMs reprogramming and suppressing immunosuppressive tumor microenvironment. We also discuss about several ways to improve the efficacy of TAM-targeting therapy from the perspective of combination therapy and specificity of TAMs subgroups.

## Introduction

In the tumor microenvironment (TME), tumor-associated macrophages (TAMs) are abundant and range from 30 to 50% of stromal cells [1]. According to the binary polarization concept, TAMs are classified to two major macrophage subpopulations, including M1-like and M2-like TAMs [2, 3].

M1-like TAMs are classical macrophages expressing molecules including NOS2, CITA and interleukin (IL)-12B, which are activated by interferon-γ, toll like receptors (TLRs), lipopolysaccharide and granulocyte macrophage colony stimulating factor [4]. M1-like TAMs strengthen T-helper 1 response and secrete pro-inflammatory cytokines including TNF-α, IL-1, IL-6, IL-12 and IL-23 [5, 6]. M2-like TAMs are alternative macrophages expressing Arg1, Chi313 and Retnla, which are activated by IL-4 and IL-13. M2-like TAMs enhance the T-helper 2 response, participate in the regression of inflammation and wound healing by secreting anti-inflammatory factors such as IL-10 and TGF-β [7]. Usually M1-like TAMs have anticancer effects, while M2-like TAMs contribute to tumor progression to some extent.

As an indispensable member of tumor tissues, TAMs promote the malignant progress of tumors in the process of interaction with cancer cells. In general, when the interactions between macrophage polarization and tumor cell plasticity reach a stable state, tumor cells will be inhibited or even disappear[8]. In view of the number of M2-like TAMs and their roles in promoting cancer, it is extremely important to understand molecular mechanism of TAMs for developing anti-cancer strategies targeting TAMs. Furthermore, TAM-targeting therapy and its combination with other anti-cancer therapies will be likely to achieve better clinical efficacy. In this review, currently available and possible future treatment options for cancer by targeting TAMs are summarized and discussed.

## TAM-associated factors in the TME

Colony-stimulating factor-1 (CSF-1) is highly expressed in various tumors, and it enhances the accumulation and migration of TAMs. TAMs are stimulated by CSF-1 to produce epidermal growth factor (EGF) to strengthen tumor invasion and metastasis. Meanwhile, tumor cells secrete CSF-1 to recruit more macrophages into the TME. The major mediators of CSF-1 include PI3K and one or more Src family kinases, which activate the ultimate migration and invasion signals in macrophages [9].

MicroRNAs (miRNAs) secreted from tumor cells are mediators between cancer cells and the TME, and there is an intercellular transfer of miRNAs between tumor cells and TAMs. Generally, the delivery of miRNAs is mainly carried out via the release and uptake of extracellular vesicles (EVs) and exosomes. EVs are released by cells to present in body fluids, enabling cells to exchange proteins, lipids and genetic materials, which plays a vital role in cell-to-cell communications [10]. There are increasing evidences that miRNAs could induce the recruitment and reprogramming of TAMs [11]. For example, colorectal cancer-derived EVs transfer miRNA-145 into TAMs, which induces TAMs to develop toward the M2 type [12]. Similarly, TAMs who absorb miR-1246-enriched exosomes trigger TAMs reprogramming into a M2-like state [13]. MiRNA-375 from the apoptotic breast tumor cells improves CCL2 secretion from tumor cells and enhances recruitment of M2-like TAMs [14]. In addition, the tumor-derived exosomes participate in angiogenesis by stimulating adenosine A2B receptor on the surface of TAMs [15].

Autophagy plays a fundamental role in controlling cell homeostasis and affects biological functions of TAMs. The damaged and excessive autophagy accelerates cell death, and ferroptosis is a form of autophagy-dependent cell death [16]. The enriched ferroptosis correlates with progressive malignancy, an aggravated immunosuppression and poor outcomes. TAMs have been confirmed to participate in ferroptosis-mediated immunosuppression [17]. The oncogenic KRAS-expressing exosomes from ferroptosis cells have been shown to induce polarization of M2-type TAMs in the TME [18]. The study indicates that targeting ferroptosis could be a strategy to regulate TAM’s polarization.

Moreover, metabolites released by cancer cells are also critical for regulating the phenotype of TAMs in the TME. For instance, succinic acid activates the PI3K/HIF-1a pathway to induce polarization of M2-like TAMs through binding to the specific membrane receptor SUCNR1 of TAMs [19]. Hence, targeting succinate and SUCNR1 might be another effective anticancer strategy by targeting TAMs in the future. Adenosine, a metabolite of a process in which ATP is converted to AMP, stimulates adenosine receptors to polarize mouse myeloid cells into an immunosuppressive phenotype [20]. The latest research shows tumor-derived adenosine facilitates cell proliferation of M2-like TAMs [21].

## TAMs enhance cancer progression

**Fig. 1:**
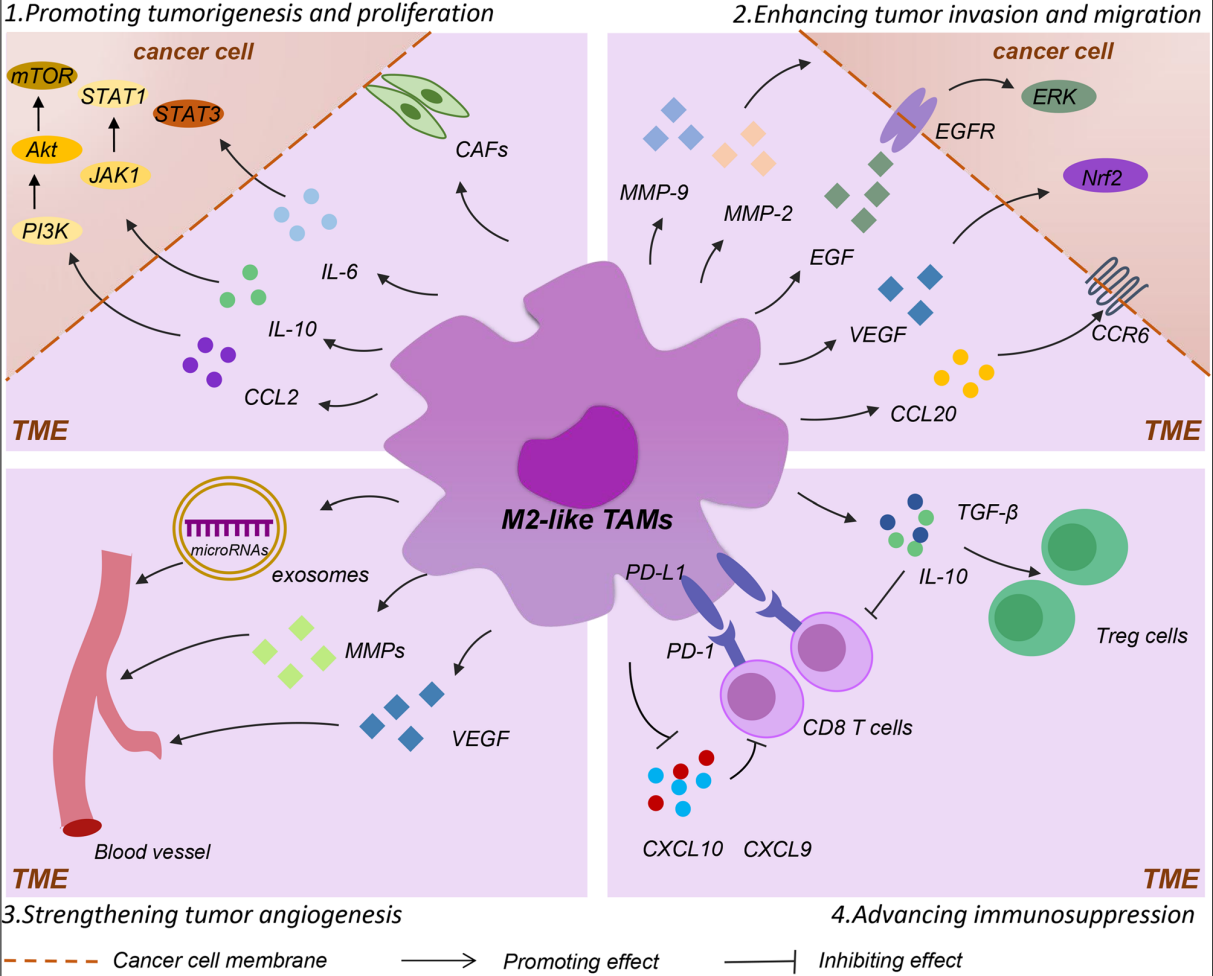
M2-like TAMs enhance cancer progression. M2-like TAMs accelerate cancer progression through four ways. (1) M2-like TAMs promote cell proliferation and tumorigenesis via secreting growth factors including CCL2, IL-6 and IL-10 to advance the activation of proliferation pathways in cancer cells. In addition, M2-like TAMs act on CAFs to lead to ECM degradation. (2) M2-like TAMs secrete EGF/VEGF to activate EMT-related pathways, and produce MMPs and CCL20 to enhance tumor invasion and migration. (3) M2-like TAMs strengthen tumor angiogenesis by secreting exosomes, MMPs and VEGF. (4) M2-like TAMs advance immunosuppression by downregulating CXCL9 /CXCL10 and secreting IL-10 and TGF-β to inhibit recruitment of CD8 T cells, while IL-10 and TGF-β improve cell proliferation of Tregs that contribute to tumor immune escape. *TAMs* tumor-associated macrophages, *TME* tumor microenvironment, *CAFs* tumor-associated fibroblasts, *CCL2* chemokine (C-C motif) ligand 2, *MMPs* matrix metalloproteinases, *VEGF* vascular endothelial growth factor, *PD-1* programmed cell death 1(PD-1), *PD-L1* PD ligand 1, *Tregs* regulatory T cells

TAMs enhance cancer progression in four ways, including promoting tumorigenesis and cell proliferation, enhancing tumor invasion/migration, strengthening tumor angiogenesis and advancing immunosuppression (Fig. 1).

### Promoting tumorigenesis and cell proliferation

M2-like TAMs secrete a variety of chemokines and interleukins including CCL2, IL-6 and IL-10, to enhance tumor cell proliferation. Among them, CCL2 activates the PI3K/Akt/mTOR signaling pathway to promote formation of endocrine resistance feedback loops in the TME, which improves tumor growth[22]. TAM-derived IL-6 contributes to cancer progression through IL-6/STAT3 pathway [23]. In a mouse cancer model, knockout of IL-6 delays tumor development [24]. Similarly, IL-10 produced by M2 macrophages has also been shown to play a catalytic role in the cancer development. For example, IL-10 produced by M2 macrophages promotes progression of non-small cell lung cancer through the JAK1/STAT1/NF-kB/Notch1 pathway [25].

Cancer stemness refers to the stem cell-like phenotype of cancer cells, which has been thought to play a major role in cancer development. Of note, cancer cells with characteristics of tumor stem cells cooperate with TAMs, which could further facilitate tumor occurrence [26]. In this process, TAMs enhance the stem cell-like properties of cancer cells by upregulating protein S100 calcium-binding protein A9 [27]. In addition, the interaction between M2-like TAMs and tumor-associated fibroblasts contributes to stiffness and degradation of extracellular matrix (ECM), and the stiffness and degradation of ECM are central factors to drive evolution of cancer [28].

### Enhancing tumor invasion and migration

Presently, tumor invasion and metastasis is an indicator to judge the malignancy of the tumor, and it is also one of the main reasons for poor prognosis of patients. Available evidence suggests that TAMs interacts with tumor cells to some extent. The interaction between tumors and TAMs contributes to the invasion and metastasis of tumor cells in a feed-forward manner [29].

M2-like TAMs advance cancer cell invasion and metastasis through multiple ways. For instance, M2-like TAMs secrete vital proteinases, such as matrix metalloproteinase (MMP) -2, MMP-9 and cathepsin, which trigger matrix degradation to promote tumor invasion and metastasis [30]. Moreover, M2-like TAMs mediate cancer cell invasion and metastasis by inducing the epithelial-mesenchymal transition (EMT) process. Among them, M2-like TAMs secrete EGF that is a pro-invasive factor to induce EMT of cancer cells by activating EGFR-ERK signals [31]. M2-like TAMs secrete CCL20 to activate CCR6 in cancer cells to enhance metastasis of primary melanoma [32]. Nuclear factor-like 2 (Nrf2) is a pleiotropic transcription factor that regulates cellular antioxidant response [33]. TAMs activate Nrf2 expression of cancer cells, which advances EMT of cancer cells via the paracrine VEGF [34].

It has been shown that M2-like TAM-derived exosomes deliver LncRNA AFAP1-AS1 to cancer cells, which downregulates miRNA-26a and enhances tumor metastasis [35]. Furthermore, there is an inflammatory feedback pathway between TAMs and pancreatic cancer cells that depends on Notch signaling to maintain tumor metastasis [36].

### Strengthening tumor angiogenesis

TAMs engage with angiogenesis and lymphangiogenesis [37–39]. The more M2-like TAMs are recruited and accumulated, the more they contribute to angiogenesis. M2-like TAMs produce pro-angiogenic factors, such as VEGF, to improve angiogenesis. VEGF stimulates formation of tumor blood vessel, while lack of VEGF leads to interruption of blood vessel development [40]. In a tumor xenograft model, TAMs enhance VEGFA-driven tumor angiogenesis [41]. In addition, MMPs from TAMs also govern tumor angiogenesis via promoting the degradation of substrate membrane and ECM [4]. Exosomes from M2-like TAMs increase microvessel density of tumor tissue in pancreatic ductal adenocarcinoma [42].

Notably, metabolism of TAMs controls tumor blood vessel morphogenesis and metastasis to some extent. For instance, the activation of mammalian target of rapamycin by deletion of REDD1 in hypoxic TAMs increases glycolysis, reduces glucose supply of endothelial cells, which improves formation of organized tumor blood vessels [43].

Taken together, in view of the role of TAMs in tumor angiogenesis, the “re-education” of TAMs is a new strategy for tumor angiogenesis inhibition and vascular normalization treatment.

### Advancing immunosuppression

Presently, a decrease in the number of invasive CD8 T cells is associated with an unfavorable prognosis for patients. Interestingly, TAMs directly or indirectly inactivate CD8 T cells through various mechanisms [44]. For instance, downregulation of CXCL9 and CXCL10 from M2-like TAMs inhibits recruitment of CD8 T cells to the TME [45]. Studies have shown that M2-like TAMs inhibit the function of CD8 T cells by retarding T cell proliferation and blocking T cell activation through interacting with inhibitory immune checkpoints [6].

Moreover, the regulatory T cells (Tregs) contribute to cancer immune escape by increasing formation of the inhibitory TME. There is a positive-feedback loop association between Tregs and M2-like TAMs. In this progress, M2-like TAMs activate Treg cells who are from CD4CD25 T cells, and in turn, the activated Treg cells differentiate monocytes to an M2-like phenotype [46].

M2-like TAMs express T cell immune checkpoint ligands including PDL1, PDL2, B7-1 and B7-2, which directly inhibit T cell function. M2-like TAMs also secrete several cytokines, such as IL-10 and TGFβ, which are helpful to maintain a strong immunosuppressive microenvironment by inhibiting CD4 T and CD8 T cells and inducing proliferation of Tregs [47]. Studies have shown that IL-10 in combination with TAMs may drive an immune-evasive microenvironment, which could be applied as a potential target for immunotherapeutic approach [48].

TAM’s activity seems to be the basis for immune suppression and adverse outcomes in cancer. A clinical trial has shown that the infiltration of immunosuppressive M2-like TAMs at the metastatic site inhibits clinically relevant immune responses in the metastatic TME [49].

## TAM-targeting therapy

**Fig. 2:**
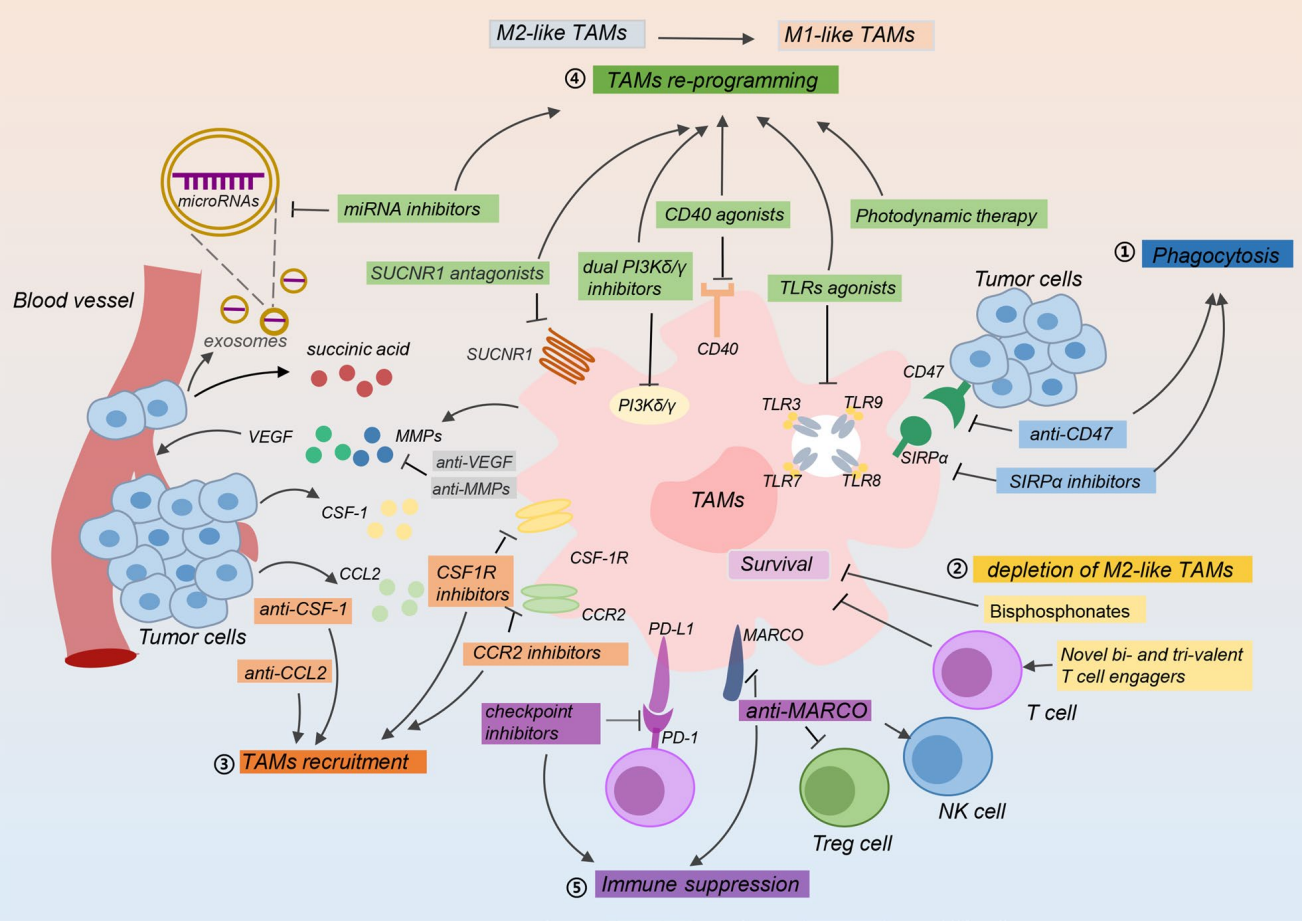
Targeting TAMs strategies in cancer therapy. Targeting TAMs for cancer treatment mainly includes five ways. (1) Promoting phagocytosis of TAMs to tumor cells. Targeting the CD47-SIRPα axis improves phagocytose ability of TAMs to tumor cells. (2) Depleting M2-like TAMs with drugs by promoting TAMs apoptosis. (3) Blocking the recruitment of TAMs to cancer cells. Drugs targeting CSF-1-CSF-1R axis and CCL2-CCR2 axis inhibit TAMs recruitment. (4) Reprograming TAMs into the M1-like type with anti-tumor activity. (5) TAMs enhance immune suppression by affecting the surrounding immune cells. Inhibiting this process is conducive to formation of immune-promoting microenvironment. SIRP*α*, signal-regulatory protein α; *CCL2* C-C motif chemokine ligand 2, *CCR2* C-C motif chemokine receptor 2, *CSF-1* colony-stimulating factor 1, *CSF-1R* colony-stimulating factor 1 receptor, *VEGF* vascular endothelial growth factor, *MMPs* matrix metalloproteinases, *SUCNR1* succinate receptor 1, *PI3Kδ/γ* phosphoinositide 3-kinase-δ/γ, *TLRs* toll-like receptors, *miRNA* microRNA, *PD-1* programmed cell death 1, *PD-L1* PD ligand 1

M2-like TAMs are potential tumor markers for a variety of cancers. Considering M2-like TAMs functions in the TME, targeting TAMs will be a potential good cancer treatment strategy. The current TAM-targeting therapies are roughly divided into five types, including promoting phagocytosis of TAMs, TAMs depletion, blocking TAMs recruitment, TAMs reprogramming and suppressing the immunosuppressive TME (Fig. 2). Anticancer drugs and candidate drugs related to TAM-targeting therapy are summarized in Table 1.

**Table 1:** TAMs related anticancer drugs and candidate anticancer drugs

Targeting TAMs strategies	Name	Target	Inhibitor type	Cancer type	Refs.	
Phagocytosis	RRX-001	CD47, SIRP-α	SMC	Non small-cell lung cancer	[83]	
	Hu5F9-G4	CD47	mAb	Advanced tumors	[52]	
Depletion of M2-like TAMs	Zoledronate	NA	SMC	Mammary carcinoma	[60, 61]	
TAMs recruitment	Pexidatintinib	CSF-1R	SMC	Advanced solid tumors	[64]	
	D2923	CSF-1R	SMC	Myelogenous leukemia	[66]	
	Emactuzumab	CSF-1R	mAb	Advanced solid tumors	[96]	
	3D185	CSF-1R	SMC	Colorectal *cancer*	[76]	
	PF-04136309	CCR2	SMC	Pancreatic cancer	[69]	
	CCX872	CCR2	SMC	Pancreatic cancer	[71]	
	CCR2i	CCR2	SMC	Cutaneous t-cell lymphoma	[106]	
	mNOX-E36	CCL2	SMC	Glioblastoma	[70]	
TAMs re-programming	R848	TLR7/8	SMC	Colorectal cancer	[80]	
	lefitolimod	TLR9	SMC	Small-cell lung cancer	[81]	
	RP6530	PI3Kδ/γ	SMC	Hodgkin lymphoma	[84]	
Suppressing the immunosuppressive TME	Anti-MARCO	MARCO	mAb	Breast and colon carcinoma	[91]	
*SMC* small molecule compound, *mAb* monoclonal antibody						

### Promoting phagocytosis of TAMs

Improving the phagocytic activity of TAMs is becoming a new way for treating cancer. The CD47-SIRPα axis reduces ability of TAMs to recognize and phagocytose tumor cells [50] (Fig. 2). CD47 is usually overexpressed on tumor cells, which contributes to immune evasion and makes TAMs hard to recognize and engulf tumor cells. Signal regulatory protein α (SIRPα) is a myeloid inhibitory receptor from myeloid cells, including monocytes, macrophages, dendritic cells and neutrophils. SIRPα binds to the cell surface ligand CD47, which restricts innate immunity [51]. The phase I clinical trial of HU5F9-G4 indicates that enhancement of macrophage phagocytosis by blocking CD47 is a promising approach for tumor therapy [52]. Moreover, PEP-20 is a new polypeptide that targets CD47 to block CD47/SIRPα interaction. One study has demonstrated that PEP-20 and its derivatives efficiently promote macrophage-mediated phagocytosis [53]. MiR-340 suppresses CD47 in pancreatic cancer cells to facilitate the phagocytic ability of TAMs [54]. Humanized AB21, a pan-allelic anti‑SIRPα antibody, targets SIRPα to promote the phagocytosis of TAMs [55].

The emergence of chimeric antigen receptor macrophages (CAR-Ms) based cell therapy provides a new perspective for improving the phagocytic ability of TAMs [56]. The CAR enhances secretion of cytokines of TAMs, polarizes TAMs to the inflammatory/anti-tumor M1 type, enhances the phagocytic function of TAMs and activity of anti-tumor cells in vivo [57]. In humanized mouse models, CAR-Ms has been further proven to induce the pro-inflammatory TME and enhance anti-tumor T cell activity [58].

### Depletion of M2-like TAMs

Selective consumption of M2-like TAMs in drug treatment effectively inhibits cancer progression. Bisphosphonates are used to treat osteoporosis, other bone diseases and several cancers. Bisphosphonates exert antitumor activity by targeting TAMs in breast cancer [59]. It is worth noting that bisphosphonates give rise to TAMs apoptosis and suppress the release of pro-angiogenic factors by inhibiting cell proliferation, migration and invasion of TAMs (Fig. 2).

Zoledronate belongs to the latest generation of bisphosphonate and is mainly used for bone metastasis of cancer. Several studies have revealed that zoledronate attacks TAMs, which leads to depletion and reprogramming of TAMs. For instance, the lipid-coated calcium zoledronate nanoparticles specifically target M2-like TAMs, reduce immunosuppressive effects and inhibit tumor growth in a tumor-bearing mouse model [60]. The preclinical evaluation confirmed zoledronate was loaded on red blood cells as a pharmaceutical preparation to remove TAMs, which is a suitable macrophage-targeted drug delivery system [61]. Moreover, novel bi- and tri-valent T cell engagers activate endogenous T cells, and specifically deplete M2-like TAMs with retaining antitumor M1-like TAMs [62]. TD-92 is a new type of erlotinib derivative, which enhances the antitumor effect of anti-PD-1 and depletes TAMs by downregulating CSF-1R [63].

Nevertheless, depletion of TAMs does not lead to a durable anti-cancer response. The anticancer effect is slightly worse than blocking TAMs recruitment and TAMs reprogramming.

### Preventing recruitment of M2-like TAMs

The recruitment of M2-like TAMs to the tumor position is influenced by several factors in the TME. Preventing the recruitment of M2-like TAMs might be a potential way for anti-cancer targeting TAMs.

#### Targeting the CSF-1/CSF-1R axis

The CSF-1/CSF-1R axis is vital to the accumulation and migration of TAMs. Consequently, blocking CSF-1R would be an effective way to prevent TAMs recruitment into tumors (Fig. 2). There are several drugs targeting CSF-1/CSF-1R in clinical trials. In a phase Ib study of PLX3397 which is efficacy for patients with advanced solid tumors, CSF-1R signaling is interrupted by PLX3397 and the M2-like TAMs is significantly reduced at tumor sites in patients [64]. In a randomised phase 3 trial, PLX3397 shows a potent antitumor response in tenosynovial giant cell tumor and ameliorates symptoms of patients [65]. The compound D2923, as a new selective inhibitor of CSF1R, has strong antitumor activity in vitro and in vivo, and it is accompanied by the depletion of M2-like TAMs in tumors [66].

Although CSF1R inhibitors are therapeutically attractive, clinical trial outcomes based on CSF1R blocking strategies have challenging in improving patient’s condition. One reason is that CSF1R inhibitors lead to accumulation of a large number of immunosuppressive cells in the tumor site. To solve this problem, CSF1R inhibitors are used in combination with CXCR2 antagonists, which blocks the tumor granulocyte recruitment and exerts a strong antitumor effect [67].

#### Targeting the CCL2/CCR2 axis

Targeting the CCL2-CCR2 axis interrupts the recruitment of monocytes to tumors, keeps monocytes in the bone marrow, reduces the number of M2-like TAMs at primary and metastatic sites, and increases CD8 T cells, finally inhibits tumor growth and invasion [68]. A phase 1b trial demonstrates that the orally administered CCR2 inhibitor PF-04136309 in combination with FOLFIRINOX chemotherapy is safe and tolerable for borderline resectable and locally advanced pancreatic cancer [69]. In a rat model of glioblastoma, the CCL2 inhibitor mNOX-E36 inhibits the recruitment of M2-like TAMs and improves the antiangiogenic treatment of glioblastoma. The clinical role of this inhibitor remains to be verified [70]. Moreover, a phase 1b study is performed for evaluating safety and efficacy of CCR2 antagonist CCX872 in patients with pancreatic cancer. CCX872 efficiently reduces tumor associated MDSCs which are converted to TAMs in the TME, and improves survival in animal models of glioblastoma [71].

#### Targeting neddylation modification

Inhibition of protein neddylation modification reduces recruitment of monocytes/TAMs. Targeting neddylation modification suppresses the intravascular survival and extravasation of tumor cells [72]. For instance, the neddylation pathway promotes activation of CCL2 and the infiltration of TAMs in lung cancer. Therefore, inactivation of the neddylation pathway interrupts the recruitment and infiltration of monocytes/TAMs in tumors, and reduces cancer cell metastasis and improves overall quality of life for patients [73]. Therefore, targeting the neddylation pathway may be a significant approach of TAMs-related anticancer therapies in future.

### TAMs reprogramming

The typing and function of TAMs are determined by the stimulation of numerous extracellular factors [6]. When TAMs accumulate in the tumor site and are stimulated by various cytokines in the TME, they gradually shift from M1-like TAMs to M2-like TAMs, which drive immune suppression and promote tumor progression. Accordingly, re-educating M2-like TAMs into M1-like TAMs, namely reprogramming TAMs, will be a new and effective anti-cancer strategy (Fig. 2).

Although CSF-1R inhibitor PLX3397 exerts anti-cancer effects by inhibiting recruitment of TAMs [64], yet it was argued that PLX3397 retards tumor growth by altering TAMs polarization rather than exhausting TAMs [74, 75]. The 3D185 is a new type of effective inhibitor blocking CSF-1R, which leads to reprogramming of TAMs and delays tumor growth in preclinical evaluation [76]. Furthermore, CSF1R-blocking abs contributes to reprogramming of TAMs for the treatment of myeloma [77].

TLRs regulate the reprogramming of TAMs. For instance, TLR3/7/8/9 improve the immune-mediated control of malignant diseases and transform M2-like TAMs to M1-like type, which limits tumor progression [78–80]. TLRs agonists are in clinical trials for tumor therapy. The TLR9 agonist lefitolimod effectively modulates the TME and induces antitumor responses by promoting infiltration of CD8 T cells and reprogramming TAMs [79]. Similarly, a phase II study on lefitolimod is evaluated the effectiveness and safety of drug maintenance therapy for small-cell lung cancer [81]. Hence, targeting TLRs of TAMs selectively may be a favorable anti-cancer way.

RRX-001, a pleiotropic anticancer drug in a phase III clinical trial, is a macrophage stimulating agent and macrophage sensitizer that enhances TAMs reprogramming [82]. It acts as a dual small molecule checkpoint inhibitor by downregulating CD47 of cancer cells and SIRP-α of monocytes/macrophages [83]. The dual PI3Kδ/γ inhibitor RP6530 inhibits proliferation of Hodgkin lymphoma cells and makes immunosuppressive M2-like phenotype toward a pro-inflammatory M1-like state [84].

Photodynamic therapy (PDT) is a treatment approach that uses cytotoxic reactive oxygen species generated by a photosensitizer under light irradiation to induce chemical damage to cause tumor cell death. PDT has been used in the clinic for more than 40 years for treating a variety of cancers [85]. Reprogramming TAMs into the anti-tumor M1-like TAMs through PDT is promising to overcome the immunosuppression of the TME [86]. Similarly, overexpression of miR-99b, miR-130 and miR-33 retards tumor progression by reprogramming M2-like TAMs to the M1 phenotype [87, 88].

### Suppressing the immunosuppressive TME

In view of various components interplaying in the TME, targeting TAMs will affect other important immune cells in the TME, which shapes an immune-promoting microenvironment. For example, targeting TAMs subgroup with expressing the scavenger receptor MARCO reverses the immunosuppressive TME by downregulating activity of Treg cells, enhancing NK cell activation and NK cell-mediated killing [89–91] (Fig. 2).

## TAM-associated therapy in combination with other anticancer regimens

A combination of several therapy reagents to treat cancer usually shows more better therapeutic effects than a single therapy recipe. TAMs-targeting therapies in combination with other anticancer therapies are summarized in Table 2.

**Table 2:** *Strategies to target TAMs in combination with other anticancer therapies*

Therapy strategies	Drug combination	Cancer type	Refs.
TAMs-chemotherapy	CSF1R blocking mAb + bortezomib	Multiple myeloma	[77]
	CSF1R blocking mAb + melphalan		
	PF-04136309 + FOLFIRINOX	Pancreatic cancer	[69]
	Emactuzumab + paclitaxel	Advanced/metastatic solid tumor	[96]
	R848 + oxaliplatin	Colorectal cancer	[80]
TAMs-immunotherapy	CCR2i + anti-PD-1	Cutaneous t-cell lymphoma	[106]
	CCX872 + anti-PD-1	Glioblastoma	[71]
	Lefitolimod + anti-PD-1	Melanoma	[79]
TAMs-nanotechnology	R848 + β-cyclodextrin	Colorectal cancer	[109]
	MiR155 + LDH	Colorectal cancer	[112]

### Combination with conventional anticancer therapies

The essential conventional treatment methods for cancers include surgery, chemotherapy and radiotherapy (RT). In addition to the TAMs-targeting therapy on cancer treatment, the combinations between TAMs and traditional chemotherapy and RT are also being explored.

The role of TAMs in chemotherapy depends on tumor types and chemotherapeutic drugs. For example, in a clinical study about ovarian cancer, the macrophages are activated after cisplatin chemotherapy, which increase CCL20 level and activate CCR6 of cancer cells to trigger EMT and reduce efficacy of chemotherapy [92]. Conversely, paclitaxel (PCX) reprograms M2-like to M1-like TAMs in a TLR4-dependent manner, which contributes to the antitumor activity of PCX [93].

M2-like TAMs reduce the efficacy of chemotherapy and increase rate of tumor recurrence, which is related to chemotherapy resistance [94]. The combined treatments with TAM-targeted drugs and conventional chemotherapeutics will reduce the adverse prognosis. Besides, blocking CSF-1R of M2-like TAMs strengthens intratumoral type I interferon release, which plays a synergistic effect with chemotherapy and enhances efficacy of platinum-based drug chemotherapy [95]. CSF1R blockers in combination with bortezomib or melphalan have shown additional therapeutic efficacy in myeloma [77]. Emactuzumab is a monoclonal antibody of CSF-1R that targets M2-like TAMs. Phase I clinical trials have shown that emactuzumab alone or in combination with PCX for patients with advanced/metastatic solid tumors lead to depletion of M2-like TAMs, which indicates that drug combination improves anti-cancer efficacy [96]. Moreover, TLR 7/8 agonists alleviate chemotherapy resistance in colorectal cancer by promoting the polarization of bone marrow-derived suppressor cells to M1-like TAMs [80].

Although RT is effective in controlling local tumors, yet ionizing radiation triggers endothelial cell damage and causes a variety of anti-tumor immune responses or immunosuppression including recruitment of M2-like TAMs. RT leads to an accumulation of bone marrow-derived M2-like TAMs in tumors. Besides, radiation-induced EMT promotes polarization of M2-like TAMs during tumor regrowth after RT [97]. When the immunosuppressive patients receive RT, M2-like TAMs will be recruited into tissues and stimulated by ionizing radiation to accelerate the recovery of blood flow in the tumor, thereby promoting tumor recurrence [98]. Thus, exclusion of M2-like TAMs after RT is an effective method to enhance tumor sensitivity to radiation and protect irradiated normal tissue [99]. In conclusion, TAM-targeting therapy with chemotherapy and RT will improve the anti-cancer efficacy of clinical chemotherapy and patient prognosis.

### Combination with novel anticancer therapies

#### Immunotherapy by immunological checkpoint inhibitors

**Fig. 3:**
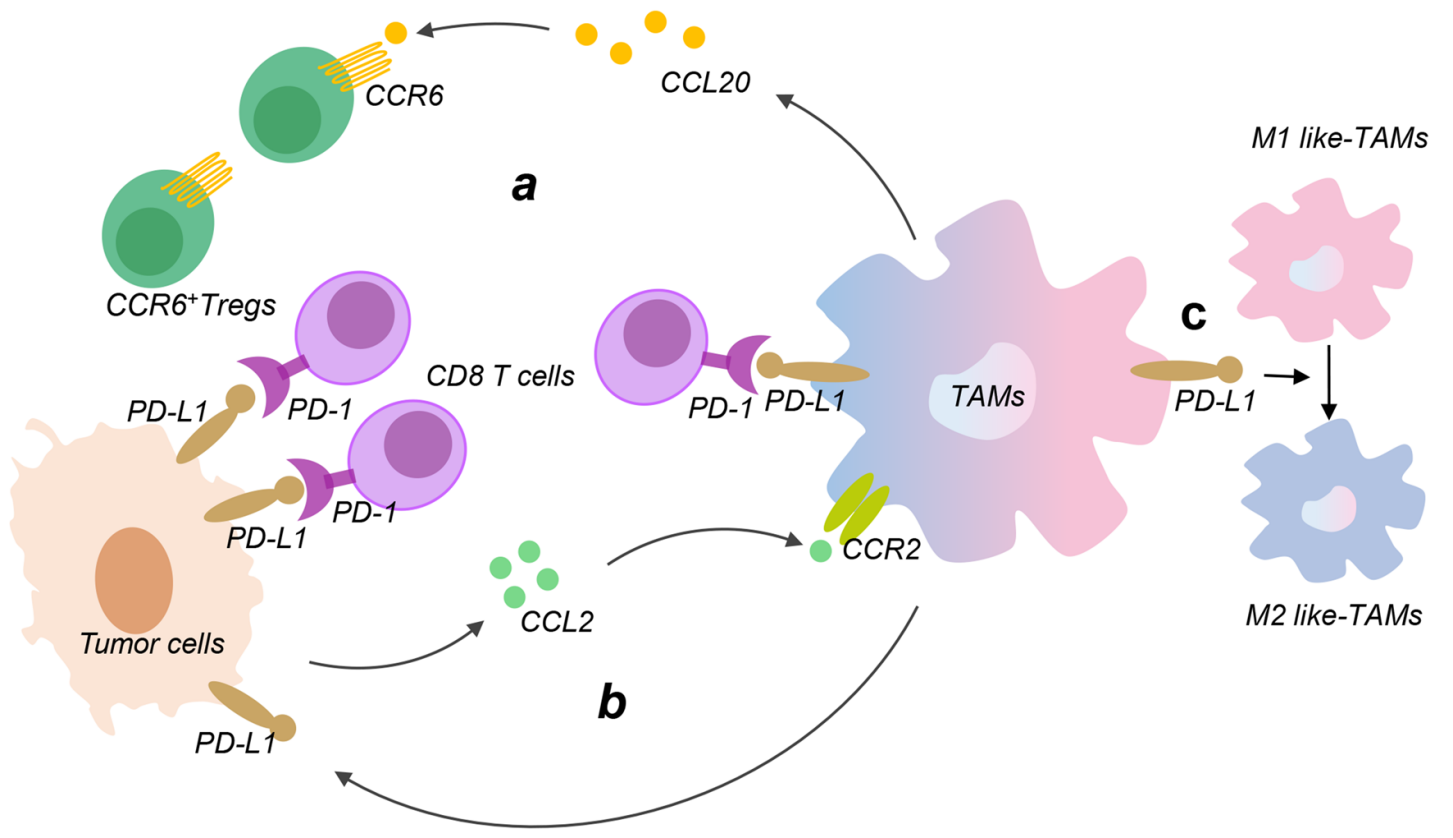
Relations between TAMs and PD-1/PD-L1 checkpoint in immune suppressive TME. **a** TAMs promote expression of CCR6^+^ Tregs by secreting CCL20, which accelerates the formation of immunosuppressive TME. Moreover, the PD-L1 of TAMs binds with the PD-1 of CD8 T cells, which inhibits ability of CD8 T cells to recognize cancer cells. **b** Tumor cells enhance the recruitment and polarization of M2-TAMs through the CCL2-CCR2 axis. Meanwhile, TAMs in turn facilitate the expression of PD-L1 in tumor cells, which forms a positive feedback loop and leads to tumor immune escape. **c** PD-L1 upregulation in TAMs enhances the polarization of TAMs from M1-like TAMs to M2-like TAMs. *PD-1* programmed cell death 1(PD-1), *PD-L1* PD ligand 1, *CCL2* chemokine (C-C motif) ligand 2, *CCR2* chemokine receptors type 2, *Tregs* regulatory T cells

PD-L1 on the surface of cancer cells is mainly responsible for helping cancer cells escape from T-cell immune surveillance. TAMs have ability to regulate PD-L1 on the tumor surface. Targeting TAMs-dependent PD-1/PD-L1 is a promising strategy to improve efficacy of PD-1/PD-L1 inhibitors (Fig. 3). The hypoxic environment upregulates PD-L1, meanwhile M2-like TAMs upregulate PD-L1 expression on tumor cells and tumor infiltrating immune cells, thereby affecting immunotherapy for non-small cell lung cancer [100]. Moreover, M2-like TAMs also affect efficacy of anti-PD-1/PD-L1 therapy by recruiting other immune cells. In hypoxic microenvironment, the TREM-1-expressing M2-like TAMs recruit CCR6-containing Tregs near liver cancer cells by secreting CCL20, which is the principal cause of resistance to anti-PD-L1 immunotherapy in liver cancer [101].

In addition to TAMs promote tumor immune escape by regulating PD-L1 on the tumor surface, the TAMs themselves express PD-L1 to contribute to tumor immune escape [102, 103]. Firstly, PD-L1 on TAMs induces immunosuppressive M2-like phenotype of TAMs. However, the phenotype will be reversed to M1 type after PD-L1 antibody treatment, which triggers macrophage-mediated anti-tumor activity [104]. Secondly, PD-L1 on TAMs may contributes to immunosuppression in the TME by interacting with PD-1 of T cells.

It is worth noting that the response rate of patients with PD-1/PD-L1 immunotherapy is only about 30% in clinical treatment. Due to the heterogeneity of PD-L1 expression in tissues, the detection result of PD-L1 expression is prone to false negatives, which is not conducive to formulating a suitable treatment plan for patients. Drugs targeting TAMs improve efficiency of anti-PD-1/PD-L1 treatment in clinical trials. New research suggests that CCL2-CCR2 axis recruits M2-like TAMs to induce immune evasion in esophageal cancer via PD-1 signaling [105]. Anti-PD-L1 drugs and small molecule CCR2 antagonists that deplete M2-like TAMs play a synergistic effect and reduce tumor survival [106]. The combination of CCR2 antagonist CCX872 and anti-PD-1 therapy further improves the survival rate of clinically relevant murine glioma models [71]. Similarly, the combination of PD-1 antibody with CSF-1R inhibitor and selective CXCR2 inhibitor significantly reduce tumor growth compared with PD-1 antibody alone. TLR9 agonist lefitolimod in combination with immune checkpoint inhibitor effectively regulates the TME and significantly enhance santitumor effect of anti-PD1/PD-L1 antibodies, which makes lefitolimod an ideal candidate for improving immune checkpoint therapy [79]. In addition, depletion of TAMs enhances local and systemic platelet-mediated anti-PD-1 delivery for postoperative treatment of tumor recurrence [107].

In conclusion, the role of TAMs in immunotherapy is of vital importance. TAM-targeting therapy in combination with immune checkpoint therapy significantly enhances immunotherapy efficiency, which may create new opportunities for tumor treatment.

#### Nanotechnology is applied to targetTAMs

Nanoparticles increase the targeting of drug delivery with minimal side effects. Recent developments of nanotechnology targeting TAMs have captured much attention. Normally, nanomaterials serve as a drug carrier delivering drugs to macrophages. Rational design of drug-nanoparticle combination effectively controls the ability of TAMs to regulate the TME, so as to achieve the purpose of targeting macrophages against cancer. As an example, a nanocarrier targeting mannose receptor of M2-like macrophages encapsulates the in vitro-transcribed mRNA encoding M1-like TAMs polarized transcription factor, which reprograms M2-like TAMs to exert anti-tumor effects without causing systemic toxicity[108]. Similarly, R848-loading β-cyclodextrin nanoparticles efficiently deliver TLR7/8-agonist to TAMs in vivo [109], which enables the drug to precisely target macrophages and elicit TAMs re-education in vivo. A micellar nanodrug effectively repolarizes M2-like TAMs to M1-like TAMs through M2-targeting codelivery of IKKβ siRNA and STAT6 inhibitor AS1517499, thereby inhibiting tumor growth and metastasis. It is worth noting that the nano-delivery system can actively target M2-like macrophages only in the acidic TME, which reduces immune side effects [110].

Furthermore, nanomaterials could directly act on TAMs to exert related anti-cancer effects. Nanoparticles promote TAMs reprogramming through interaction of their biological regulation with the intrinsic properties of TAMs. For instance, The hyaluronic acid-modified superparamagnetic iron oxide nanoparticles artificially reprogram macrophages to effectively counteract intratumoral immunosuppression, and a living cell therapy protocol is designed using a nanoparticle-assisted cell reprogramming strategy [111]. Compared with the common nanomaterial methods, therapeutic biologics on living cells reprogrammed with nanomaterials may be preferable and more advantageous to induce certain cells to produce biological responses in vivo.

The M2-like TAM-targeting nanoparticles will act on cancer cells in combination with encapsulating targeting drugs. Therefore, the nano-drug carrier and the targeting drug simultaneously play crucial role in cancer treatment. The LDH (layered double hydroxide) is a nanoparticle that upregulates myeloid proinflammatory cytokines (TNF-α, IL-12) and co-stimulatory molecules including CD40, CD80, CD86 and MHC class II. LDH nanoparticles containing with miR155 have superior ability to target M2-like TAMs, reprogram M2-like TAMs to M1 subtype and improve the TME [112].

## Exploration of heterogeneous subgroups of TAMs in the TME

All cancer therapies including TAM-targeting therapy aim to improve the prognosis of cancer patients. Currently, the efficacy of TAM-targeting therapy is not satisfactory enough. So far, improvement of TAM-targeting therapy efficacy is urgent for enhancing the prognosis of cancer patients. In addition to enhance the application of targeted therapies and combination treatments, it is also crucial to explore more heterogeneous subgroups of TAMs to serve as targets in the TME.

TAMs-associated therapy is an attractive therapeutic method to facilitate anti-tumor immune response. However, the heterogeneity of TAMs and the different cell-cell interaction patterns make the TAMs-targeting strategy variable and uncertain. Therefore, it is necessary to discover the specificity of subsets of TAMs as a basis for TAMs-targeting therapy.

Presently, single-cell RNA sequencing (scRNA-seq) is a powerful tool to determine the diversity of tumor cells. It is applied to explore the heterogeneity of TAMs and to describe the subgroups of TAMs accurately [113]. For instance, two different populations of TAMs, C1QC + TAMs and SPP1 + TAMs, are found by scRNA-seq in colon cancer. The specific depletion of SPP1 + TAMs may eventually improves myeloid-targeted immunotherapy [114]. In chronic hepatitis B and C virus-related hepatocellular carcinoma (HCC), the M2-like TAMs with high expression of CCL18 and transcription factor CREM are uncovered by scRNA-seq. The M2-like TAMs are enriched in patients with advanced HCC and might be involved in tumor progression [115].

By now, there is still numerous works to be done in order to combine single-cell resolution with clinical significance. Once these findings are transformed into measurable parameters that can be integrated into the prognostic score [116], which facilitates determination of clinically personalized treatment plans and improves accuracy of prognostic analysis.

## Conclusions and perspectives

In recent years, the application value of targeting TAMs in cancer treatment has been paid increasing attention. However, the TAMs-associated therapies still have multitude shortcomings, such as large side-effects and poor efficacy. Several drugs or therapies work well in the preclinical trials on animal models, but the results are poor in the clinical trials on patients. Moreover, the role of TAMs in cancers varies with the type of cancers and individual differences of patients are underestimated, which leads to unstable efficacy. Several challenging issues have yet to be solved, including other uncovered functions of TAMs in the TME, the best clinical combinations of TAMs-targeting therapy with other therapies and how to avoid high side-effects of TAMs associated-therapy.

Novel anti-cancer TAMs-targeting therapies are constantly being developed and improved, which is expected to break through traditional tumor-associated therapies and gains favorable clinical treatment results. Furthermore, a combination of TAM-targeting drugs with other anticancer drugs to get better efficacy will be an irresistible trend. Currently, the combination of TAM-targeting therapy with immunotherapy and nanotechnology improves the clinical anticancer efficacy. In future, targeting TAMs to fight cancer will also be an important battlefield for combination therapy.

## Data Availability

Not applicable.

## Abbreviations

TME: Tumor microenvironment
TAMs: Tumor-associated macrophages
MMPs: Matrix metalloproteinases
EGF: Epidermal growth factor
EMT: Epithelial-mesenchymal transition.
VEGF: Vascular endothelial growth factor.
Tregs: Regulatory T cells.
CSF-1: Colony-stimulating factor-1
miRNAs: microRNAs
EVs: Extracellular vesicles
ECM: Extracellular matrix
Nrf2: Nuclear factor-like 2
CAR-Ms: Chimeric antigen receptor macrophages
TLRs: Toll-like receptors
RT: Radiotherapy
PCX: Paclitaxel
PD-1: Programmed cell death 1(PD-1)
PD-L1: PD ligand 1
LDH: Layered double hydroxides
scRNA-seq: Single-cell RNA sequencing
HCC: Hepatocellular carcinoma
